# Bacterial aortic endarteritis with sepsis as a rare complication of aortic dissection in a cat

**DOI:** 10.1093/jvimsj/aalag243

**Published:** 2026-10-07

**Authors:** Sukjung Lim, Kristen L Shekelle, Katia R Groch, Brian F Porter, Elizabeth Malcolm, Sonya Gordon

**Affiliations:** Department of Small Animal Clinical Sciences, College of Veterinary Medicine and Biomedical Sciences, Texas A&M University, College Station, TX 77843, United States; Department of Veterinary Pathobiology, College of Veterinary Medicine and Biomedical Sciences, Texas A&M University, College Station, TX 77843, United States; Department of Veterinary Pathobiology, College of Veterinary Medicine and Biomedical Sciences, Texas A&M University, College Station, TX 77843, United States; Department of Veterinary Pathobiology, College of Veterinary Medicine and Biomedical Sciences, Texas A&M University, College Station, TX 77843, United States; Angell Animal Medical Center, Boston, MA 02130, United States; Department of Small Animal Clinical Sciences, College of Veterinary Medicine and Biomedical Sciences, Texas A&M University, College Station, TX 77843, United States

**Keywords:** aortic dissection, infective aortic endarteritis, *Pasteurella*, systemic hypertension

## Abstract

A 14-year-old domestic shorthair cat was examined because of respiratory distress. Thoracic radiography showed a prominent aortic arch and no other abnormalities. Echocardiography documented a mobile flap within the ascending aorta creating 2 lumens, consistent with aortic dissection (AD), mild pericardial effusion with tamponade, left ventricular concentric hypertrophy, and normal left atrial size. Systemic hypertension (200 mmHg) was the presumed cause of AD, prompting administration of telmisartan, furosemide, and amlodipine. The cat clinically improved, became normotensive, and was discharged 2 days later. The cat remained well for 4 months, then became lethargic and died. Necropsy showed a 7.6 cm long aortic intimal flap, severe bacterial aortic endarteritis, and evidence of sepsis including multiple organ necrosis, thrombosis, and embolic glomerulonephritis. Bacterial culture of the kidneys yielded mixed bacterial growth including *Pasteurella stomatis*, presumably the same bacterium identified in the endarteritis lesion. Infective aortic endarteritis can be a fatal complication of AD.

## Case report

A 14-year-old, 6.1 kg, spayed female domestic shorthair cat ​presented for an acute onset of open mouth breathing and lethargy. The cat had no previous relevant medical history​. The cat had been vaccinated against common pathogens including feline leukemia virus. The cat was quiet alert and responsive. The heart rate was 220 beats per minute and the respiratory rate was 40 breaths per minute with mildly increased respiratory effort. A grade IV/VI left parasternal systolic heart murmur was auscultated. The rectal temperature was 100.8 F. The cat was obese with a body condition score of 8/9. An oral examination was unable to be performed due to temperament. The remainder of the physical examination showed no abnormalities. Indirect arterial systolic blood pressure was 140 mmHg by Doppler sphygmomanometry. Three-view thoracic radiographs showed a prominent bulge in the region of the ascending aortic arch. No obvious cardiac or pulmonary abnormalities were noted. Transthoracic echocardiography showed a partially mobile linear flap within the ascending aorta with a true aortic lumen in the center and false lumen outside of the flap (Figure 1). There was systolic expansion of the flap and diastolic collapse of the flap. The flap extended from of the aortic annulus to the aortic arch, although the distal end of the flap was not well visualized. A diagnosis of Stanford type A aortic dissection (AD) was made based on these findings.^1^ Color Doppler imaging of the aorta showed systolic flow from the true lumen to the false lumen (Figure 2). There was systolic turbulent blood flow in the true lumen starting immediately proximal to the dissection flap with a dynamic flow profile noted on spectral Doppler with a peak velocity of 1.85 m/s. The aortic root was subjectively dilated with mild aortic regurgitation. There was diffuse left ventricular concentric hypertrophy with a maximal left ventricular wall thickness of 7.1 mm on 2D measurements (body-weight adjusted reference range, 3.1-5.4 mm).^2^ The left ventricular end-diastolic and end-systolic dimensions were 14 and 3.8 mm, respectively, with a left ventricular fractional shortening of 73%. There was mild pericardial effusion with cardiac tamponade evidenced by diastolic right atrial wall collapse. Systolic anterior motion of the chordae tendineae of the anterior mitral leaflet with trace mitral regurgitation was noted. The mitral valve was structurally normal. The ratio of left atrial to aortic diameter measured from the right parasternal short axis view was 0.9 (reference range, 0.89-1.44).^2^ The left atrial appendage was subjectively normal in size. The maximum left atrial appendage flow velocity was 0.94 m/s (reference range, 0.47 ± 0.2 m/s).^3^ The remainder of the echocardiogram findings showed no abnormalities. A complete blood count was showed no abnormalities. A serum biochemistry panel showed mild hyperglycemia (184 mg/dL; reference range, 83-112) and mild hypokalemia (3.1 mmol/L; reference range, 3.7-5.8 mmol/L) with no other abnormalities. Urinalysis showed no abnormalities with a specific gravity of 1.022 and no proteinuria or leukocyturia. Serum thyroxine concentration was normal (2.32 μg/dL; reference range, 0.78-3.82). For initial stabilization, although the initial blood pressure measurements fell within the prehypertensive range (140-159 mmHg),^4^ anti-hypertensive treatment with telmisartan (1.6 mg/kg, PO, q12h) and furosemide (1 mg/kg, IV, q12h) was started given the presence of cardiac changes associated with hypertensive cardiomyopathy, including left ventricular concentric hypertrophy and AD. The cat was also placed in an oxygen kennel at 40% oxygen as a precautionary measure due to mild tachypnea and dyspnea. These respiratory abnormalities were presumed to be secondary to discomfort and pain associated with AD as no other apparent cause was identified. The next morning, the systemic blood pressure had increased to 200 mmHg, prompting addition of amlodipine besylate (0.1 mg/kg, PO, q24h). Oxygen supplementation was discontinued as the cat’s respiratory status had become stable. The following day, the cat was bright, alert, and responsive. The blood pressure was 143 mmHg. Furosemide was discontinued. A point-of-care blood gas and chemistry analysis (CCX, Stat Profile Critical Care Xpress 12, Nova Biomedical Corporation, Waltham, MA) showed mild hypokalemia (3.34 mmol/L; reference range, 3.4-5.5 mmol/L) with no other abnormalities. The cat was discharged from the hospital with amlodipine (0.1 mg/kg, PO, q24h) and telmisartan (1.6 mg/kg, PO, q12h).

**Figure 1:**
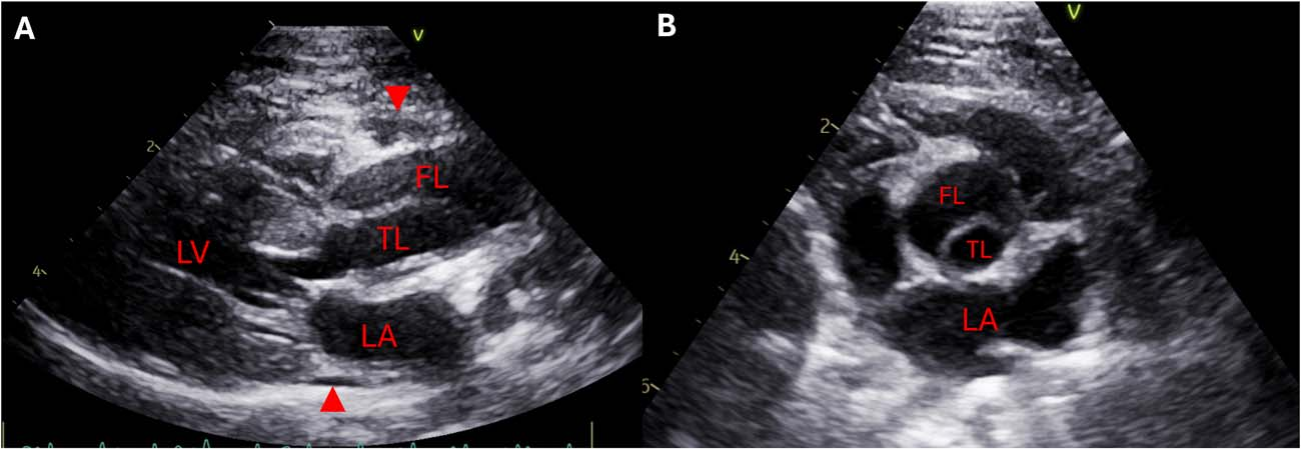
Transthoracic echocardiography images in a cat with aortic dissection. Right parasternal long-axis 5 chamber view (A) and right parasternal short-axis view optimized for aorta and left atrium (B) showing a floating aortic intimal flap dividing the dilated aorta into a true lumen and a false lumen and mild pericardial effusion (arrowhead). Abbreviations: FL = false lumen; LA = left atrium; LV = left ventricle; TL = true lumen.

**Figure 2:**
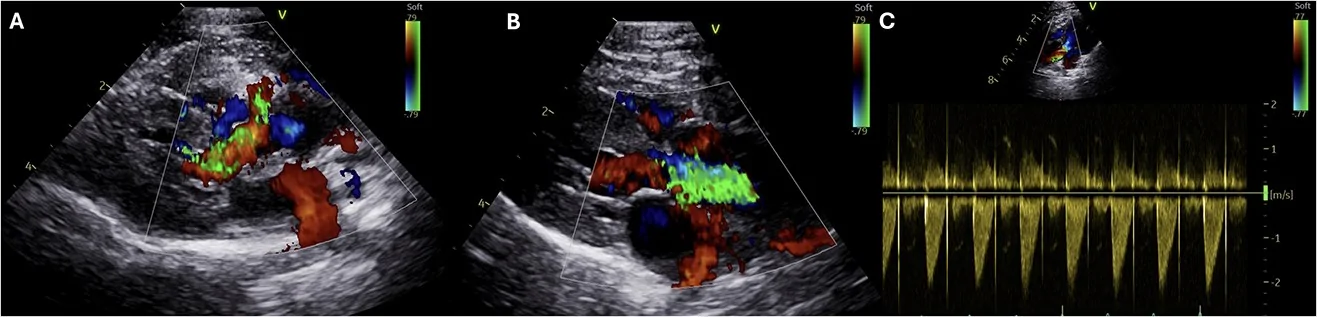
Echocardiogram findings in the same cat shown in Figure 1 (A) Color Doppler imaging showing systolic flow from the true lumen to the false lumen on right parasternal long-axis 5 chamber view. (B) One month after the diagnosis of aortic dissection, systolic flow is appreciated only within the true lumen. (C) Spectral Doppler assessment showing a mildly increased transaortic flow velocity (2.3 m/s) due to the narrowing of the true aortic lumen caused by the dissection flap.

At a 1-month follow-up visit, the owner reported that the cat had been doing well at home with no overt clinical signs. On exam, the cat was bright, alert, and responsive. Physical examination findings were largely static compared to the previous visit. The blood pressure was 180 mmHg by Doppler. An abbreviated transthoracic echocardiography was performed. Color Doppler imaging showed systolic turbulent blood flow only within the true lumen of AD, and the peak blood flow velocity in this region was 2.3 m/s on spectral Doppler assessment (Figure 2). The previously noted pericardial effusion had been resolved. The echocardiogram findings were otherwise similar compared to the initial echocardiogram. The amlodipine dose was increased to 0.2 mg/kg, PO, q24h due to persistent systemic hypertension. The cat was re-evaluated again about 1 month after the second visit, and the owner reported that the cat had demonstrated no clinical signs at home. The blood pressure was 144 mmHg by Doppler. An abdominal ultrasonography was performed to investigate abdominal diseases that could contribute to systemic hypertension. No abnormalities were identified, and both kidneys appeared structurally normal. Given the absence of an identifiable cause for systemic hypertension, the cat’s systemic hypertension was considered idiopathic.^4^ The owner was instructed to return for re-evaluation in 6 months. About 2 months after the third visit (4 months after the initial presentation), the owner reported that the cat had become progressively lethargic and anorexic. The cat was found dead at home about 1 week later.

A necropsy was performed approximately 8-14 h after death. Gross findings included a defect in the aortic wall 2.3 cm distal to the aortic valve that extended 7.6 cm along the descending aorta as a blind-ended lumen (dissection). The dissection was filled with dark red, gelatinous fluid (blood clot), and bulged into the vessel lumen, encompassing at least 50% of the luminal aortic diameter (Figure 3). The aortic root was mildly dilated. The left ventricular wall thickness was 10 mm, and the right ventricular wall thickness was 4 mm. The left auricle and atrium were moderately dilated. Both kidneys had numerous dark red, flat, round foci scattered throughout the cortices, consistent with petechiae. Histologic examination of the aortic dissection showed tunica media degeneration and fibrosis with granulation tissue, all of which support chronicity of the dissection, as well as acute necrosuppurative endarteritis. The lumen of the dissection was occluded by a thrombus of fibrin and numerous intralesional gram-negative coccobacilli (Figure 4). Arterioles within the left ventricle myocardium had mild tunica media hypertrophy with hyalinized walls and focal recanalization, which is consistent with systemic hypertension. There was also an area of acute cardiomyocyte degeneration and necrosis. Histology of the kidneys showed acute fibrinosuppurative glomerulonephritis with intralesional gram-negative coccobacilli and necrosis consistent with embolic glomerulonephritis (Figure 4) likely from hematogenous spread of bacteria from the aortic endarteritis. The kidneys also had chronic lymphoplasmacytic interstitial nephritis with tubular proteinosis, glomerulosclerosis, and interstitial fibrosis, changes consistent with chronic renal disease. Additional histologic findings included adrenal gland necrosis, acute pulmonary thrombosis, and multibrain necrosis. The final histologic diagnosis was severe bacterial aortic endarteritis with AD, sepsis, and possibly disseminated intravascular coagulation. Aerobic bacterial culture of kidney tissue yielded mixed growth, with notable 1+ growth of *Pasteurella stomatis* and *Actinomyces canis*. Given the gram-negative coccobacilli within the aorta and kidney histologically, the causative bacterium was most likely *P. stomatis*.

**Figure 3:**
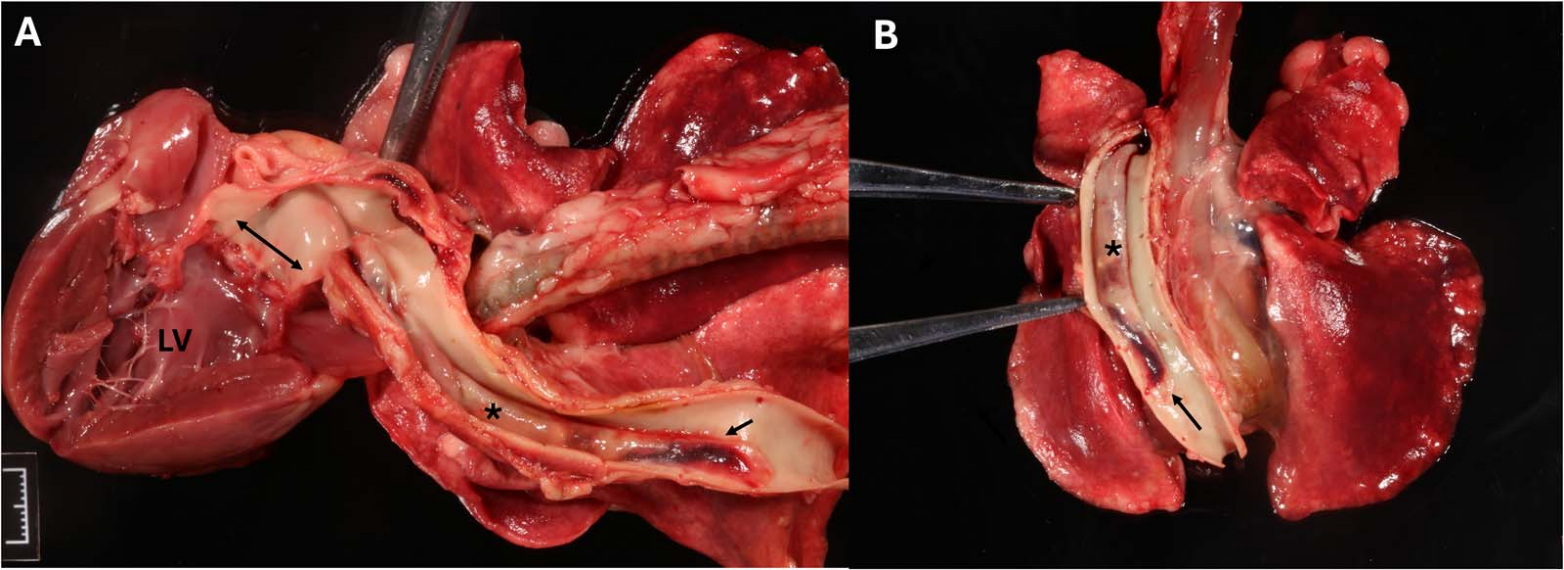
(A, B) Gross necropsy images from a cat with aortic dissection and bacterial aortic endarteritis. The defect created a blind-ended false lumen (*) that extended 7.6 cm along the descending aorta. The false lumen was filled with blood clot (arrow). The aortic root was dilated (double-sided arrow) secondary to systemic hypertension. Abbreviation: LV = left ventricle.

**Figure 4:**
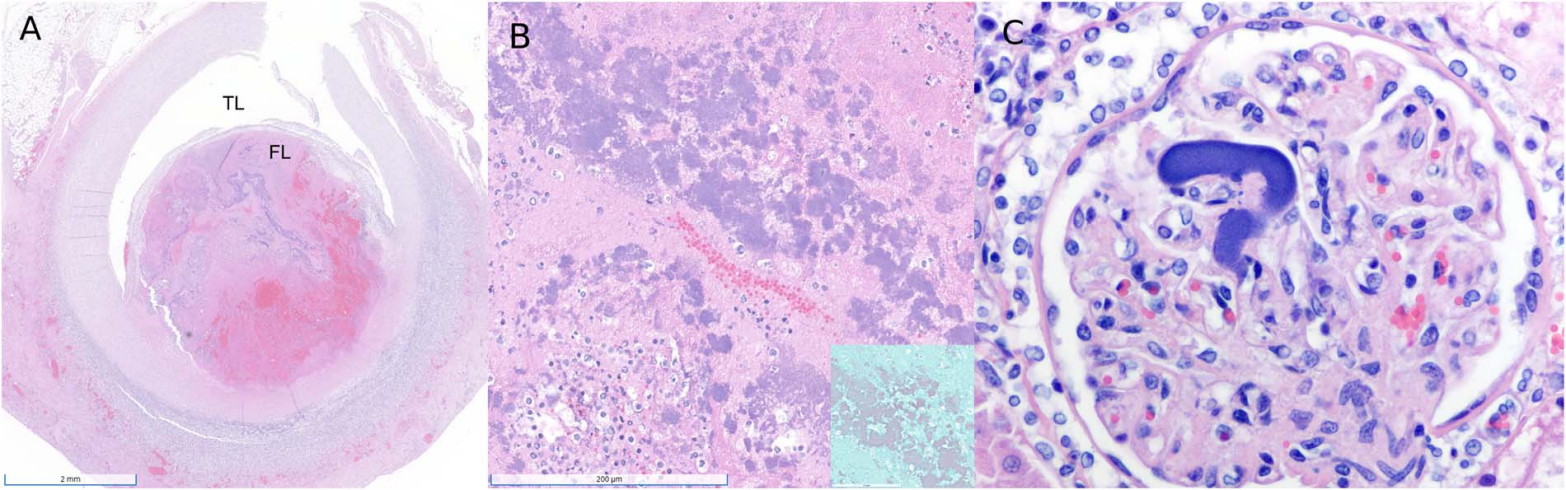
(A) Aorta at the level of dissection (1×, Hematoxylin and eosin (H&E) stain) showing extensive dissection of the tunica media with the presence of a thrombus and bacteria in the false lumen. (B) The thrombus within the false lumen (20×) is composed of fibrin, red blood cells, necrotic cellular debris, and abundant bacteria colonies. Inset (40×): The bacteria within the thrombus are gram-negative coccobacilli. (C) Severe embolic glomerulonephritis (40×, H&E stain) with bacteria within a glomerular capillary. The bacteria have the same morphology as those in the aortic thrombus. Abbreviations: FL = false dissection lumen; TL = true aortic lumen.

## Discussion

AD is a rare but fatal condition characterized by partial detachment of the inner layer (aortic intima) of the aorta, allowing blood to flow into the middle layer of the aorta (aortic media). The Stanford classification system is commonly used to categorize dissections based on their anatomic location.^1^ Type A dissections involve ascending aorta regardless of the site of the primary intimal tear, which was the type identified in this cat. Stanford type B dissections originate distal to the left subclavian artery and involve only the descending aorta.^1^ The incidence of AD in humans is 5-30 cases per 1 million people^1^ while the incidence in animals is unknown, with only several case reports in the literature. Risk factors for AD in humans include systemic hypertension, vasculitis, aortitis, blunt chest trauma, aortic aneurysm, previous surgery of the aorta, and genetic conditions, including connective tissue disorders and abnormal aortic valve or aorta.^1^^,^^5^ In animals, similar etiologies are reported. A unique veterinary etiology is *Spirocerca lupi* infection.^6^ Complications of AD in humans include aneurysm formation, aortic rupture, myocardial infarction, congestive heart failure, redissection, and death.^1^ Infective aortic endarteritis or aortitis has rarely been reported in association with AD in humans^5^^,^^7–10^ and in one animal.^11^ However, the clinical presentations described in those reports differed from that of the present report. In some of the cases, patients were diagnosed with both conditions concurrently,^5^^,^^7^^,^^9^^,^^11^ suggesting that aortic endarteritis likely contributed to AD, as it is a known risk factor. In 2 cases, the patients had a history of multiple cardiovascular diseases in addition to AD and underwent surgical repair of AD before developing aortic endarteritis as a post-operative complication.^8^^,^^10^ In contrast, the cat of this report was first diagnosed with AD and subsequently developed bacterial aortic endarteritis despite having no risk factors for infection of the aorta such as previous surgery of the aorta, congenital malformations affecting aorta, and atherosclerosis,^12^ suggesting that AD likely predisposed the cat to aortic endarteritis. To the authors’ knowledge, this unique sequence of 2 rare conditions has not been reported in either humans or animals. The authors could not exclude the possibility that endarteritis preceded the AD based solely on the postmortem findings; however, this was considered less likely, as there were no previous clinical signs or hematologic abnormalities indicative of infection before the diagnosis of AD.

Regarding the pathophysiologic mechanism for development of aortic endarteritis in the present case, it is plausible that the turbulent blood flow in the aorta secondary to true lumen narrowing caused by AD (Figure 2) might have increased the shear stress on the already damaged endothelium and disrupted intimal tissue, thereby predisposing it to bacterial adherence and subsequent infection. While this seemingly plausible complication of AD seems logical, it is most likely exceedingly rare because immediate surgical intervention is performed to repair the damaged aorta in humans with Stanford type A AD. Furthermore, in animals, AD is rare to begin with and is usually fatal with short survival times,^7^ which might further explain the rarity of this complication. Nevertheless, this complication is clinically relevant because supportive care is typically instituted in animals with AD, and clinicians should be aware of it. Additionally, it might be prudent to consider prophylactic antibiotic therapy and possibly antithrombotic therapy to minimize the risk of this complication.

*Pasteurella* species are commensal organisms of the feline oral cavity. Even though the oral examination was not performed in the cat due to the temperament, subclinical gingivitis is common in older cats, which could have provided an entry point for the bacteria, although other routes are possible. *Pasteurella* species have been reported to be responsible for dissecting aortitis in a goat^11^ and endocarditis and sepsis in humans following a cat bite or scratch.^13^^,^^14^ It is unknown if this cat had underlying immunosuppressive diseases such as feline immunodeficiency virus and feline leukemia virus infections, as testing for these infections was not performed; however, the cat had been vaccinated against feline leukemia virus, and the cat’s complete blood count results showed no abnormalities. The presence of small thrombi in multiple organs and evidence of embolic glomerulonephritis suggest hematogenous seeding of the infective thrombi from the aortic endarteritis, causing sepsis and multiple organ failure.

Systemic hypertension is the most common risk factor for AD in humans^1^ and has also been reported in cats with AD.^15^ Since AD does not commonly occur in cats with systemic hypertension, other predisposing factors might play a role including chronic degeneration of the tunica media, which was noted in 2 of 4 cats of the previous case report^15^ and also in this cat. The cat had a normal blood pressure on presentation. Although systemic blood pressure is commonly elevated in acute AD, it can be normal or even low due to impending aortic rupture, cardiac tamponade, and poor perfusion.^1^ Compression of the true lumen caused by the dissection flap can also reduce cardiac output, resulting in hypotension.^15^ In the previous case report,^15^ blood pressure was normal on presentation in all 4 cases.^15^ Cardiac tamponade was noted in 3 of 4 cats in that report and in this cat. The cat’s systemic hypertension was considered idiopathic. Although histologic evidence of chronic renal disease was present, blood biomarkers of kidney function were normal, suggesting that the systemic hypertension was unlikely to be secondary to kidney disease. Measurement of serum and urine aldosterone concentrations could have been considered to rule out hyperaldosteronism, especially since the cat’s potassium was mildly decreased. Measurement of catecholamine concentrations would have been helpful as well to rule out pheochromocytoma although an adrenal gland mass was not identified on ultrasonography or at necropsy. The left ventricular concentric hypertrophy was attributed to systemic hypertension based on the absence of typical histopathologic features of hypertrophic cardiomyopathy. Histopathologic examination of an eye showed no evidence of hypertensive vascular changes.

In summary, AD can occur secondary to systemic hypertension, however blood pressure on initial presentation can be normal or low. Bacterial aortic endarteritis can be a fatal complication of AD. The short survival time of the cat in this report aligns with previously reported poor prognosis and short survival in cats with AD.

## Off-label antimicrobial declaration

Authors declare no off-label use of antimicrobials.

## Institutional animal care and use committee or other approval declaration

Authors declare no institutional animal care and use committee or other approval was needed.

## Abbreviations

AD: aortic dissection
